# Enhanced production of a single domain antibody with an engineered stabilizing extra disulfide bond

**DOI:** 10.1186/s12934-015-0340-3

**Published:** 2015-10-09

**Authors:** Jinny L. Liu, Ellen R. Goldman, Dan Zabetakis, Scott A. Walper, Kendrick B. Turner, Lisa C. Shriver-Lake, George P. Anderson

**Affiliations:** Naval Research Laboratory, Center for Bio/Molecular Science and Engineering, Washington, DC, 20375 USA

**Keywords:** Camelid, Single domain antibody, Disulfide bond, Thermal stability, Protein production

## Abstract

**Background:**

Single domain antibodies derived from the variable region of the unique heavy chain antibodies found in camelids yield high affinity and regenerable recognition elements. Adding an additional disulfide bond that bridges framework regions is a proven method to increase their melting temperature, however often at the expense of protein production. To fulfill their full potential it is essential to achieve robust protein production of these stable binding elements. In this work, we tested the hypothesis that decreasing the isoelectric point of single domain antibody extra disulfide bond mutants whose production fell due to the incorporation of the extra disulfide bond would lead to recovery of the protein yield, while maintaining the favorable melting temperature and affinity.

**Results:**

Introduction of negative charges into a disulfide bond mutant of a single domain antibody specific for the L1 antigen of the vaccinia virus led to approximately 3.5-fold increase of protein production to 14 mg/L, while affinity and melting temperature was maintained. In addition, refolding following heat denaturation improved from 15 to 70 %. It also maintained nearly 100 % of its binding function after heating to 85 °C for an hour at 1 mg/mL. Disappointingly, the replacement of neutral or positively charged amino acids with negatively charged ones to lower the isoelectric point of two anti-toxin single domain antibodies stabilized with a second disulfide bond yielded only slight increases in protein production. Nonetheless, for one of these binders the charge change itself stabilized the structure equivalent to disulfide bond addition, thus providing an alternative route to stabilization which is not accompanied by loss in production.

**Conclusion:**

The ability to produce high affinity, stable single domain antibodies is critical for their utility. While the addition of a second disulfide bond is a proven method for enhancing stability of single domain antibodies, it frequently comes at the cost of reduced yields. While decreasing the isoelectric point of double disulfide mutants of single domain antibodies may improve protein production, charge addition appears to consistently improve refolding and some charge changes can also improve thermal stability, thus providing a number of benefits making the examination of such mutations worth consideration.

**Electronic supplementary material:**

The online version of this article (doi:10.1186/s12934-015-0340-3) contains supplementary material, which is available to authorized users.

## Background

Single domain antibodies (sdAb), the recombinantly expressed variable region from the unconventional heavy chain only antibodies found in camelids, are renowned for their properties of high affinity coupled with the ability of most to refold into an active form after denaturation [1–4]. These properties have made sdAb attractive reagents for biotechnology and medical applications, where high affinity and stable reagents are advantageous [5–9]. Although many sdAb naturally have melting temperatures above 70 °C, the majority melt at lower temperatures. Regardless of their melting temperatures, most sdAb are found to refold substantially when evaluated by circular dichroism (CD) [3, 10, 11]. When heated above their melting temperature at high concentration for longer periods of time, however, some sdAb are prone to aggregation [12, 13]. Therefore our current approach towards engineering the most rugged and robust sdAb recognition reagents is to increase both their melting temperature and solubility [13].

Structurally sdAb are homologous to the variable heavy domains from conventional antibodies, and include hypervariable regions (complementarity determining regions, CDRs) that mediate the interaction with antigen and framework regions (FRs) that forms a β sheet structure and serves as a scaffold for displaying the CDRs for antigen binding. The introduction of a disulfide bond between framework regions 2 and 3 is a demonstrated strategy for increasing the melting temperature of sdAb [14–16]. It has also proven a route towards enhancing protease resistance of the binding elements [17]. Unfortunately, this improvement can come at the expense of protein production [17–19]. This issue is dramatically demonstrated by clone L1-G2, a sdAb specific for the L1 antigen of the vaccinia virus [18]. Clone L1-G2+, a version of the L1-G2 sdAb with an additional inter-framework disulfide bond, showed a nearly 20 °C increase in its melting temperature. However the L1-G2+ clone lost its ability to refold after heat denaturation and protein production plummeted at least 5-fold from ~20 mg/L down to ~4 mg/L.

It has been established that the primary sequence of proteins is tied to their expression levels and solubility; this is seen both looking at the tendency of *Escherichia coli* proteins to aggregate as well as examining the expression of recombinant proteins in vivo [20, 21]. Other researchers, as well as ourselves, have shown that increasing the charge of recombinantly expressed antibody binding regions can increase their solubility [12, 13, 22–27]. In particular adding negative charges can lead to sdAb derived from camelid heavy chain antibodies as well as ones derived from human variable heavy domains to refold nearly 100 % after heating. We have added negative charges to sdAb as both negative tails as well as the introduction of point mutations based on the consensus sequence analysis [12, 13]. In the current work we introduced negative charges into the L1-G2+ sequence and showed that the protein production increased approximately 3.5-fold to ~14 mg/L while the melting temperature remained high, and the ability to refold was partially restored. The charged mutant with the extra disulfide had similar affinity for the L1 antigen as L1-G2+ and maintained over 90 % of its antigen binding ability up to its melting temperature of ~80 °C. We then sought to confirm this method with additional sdAb, but with only minimal success. However, we did find that negative charge addition not only improves refolding but can even result in thermal stabilization equal to disulfide bond addition, but without the negative impact on production.

## Results and discussion

The sdAb clone L1-G2 was selected from a phage display library derived from llamas immunized with both killed vaccinia virus and the L1 antigen of vaccinia [18]. This clone produced well in *E. coli* with typical yields of ~20 mg/L and showed the desired specificity for L1 and high affinity of ~1 nM, however it unfolded at 62 °C as determined by CD (Table 1), which while fairly typical for sdAb was insufficient to meet our goal of sdAb that remain stable upon exposure to a sustained temperature of 70 °C.

**Table 1:** Number of charged residues, calculated/measured isoelectric point, and Tm for sdAb

Clone	Tm (°C) Dye melt	Tm (°C) CD	Refolding (%)	Yield (mg/L)^a^
L1-G2	64	62	55	24.6 ± 5.1 (3)
L1-G2+	81	78	15	3.9 ± 1.1 (7)
L1-G2+neg	81	78	70	13.8 ± 1.4 (4)
L1-G2+neg2	81	78	78	7.9 (2)
A3	86	85	73	≥11.5 (4)
A3+	87	>90	94	1.7 ± 0.8 (4)
A3+neg	85	87	87	2.3 ± 0.7 (4)
AC	73	74	63	24.5 (2)
ACneg	80	81	87	24 (2)
ACneg2	81	80	88	17 (2)
AC+	76	82	69	3.2 ± 0.6 (6)
AC+neg	82	86	82	4.1 ± 0.5 (6)
AC+neg2	81	85	84	4.2 ± 0.5 (6)

^a^Average yield ± SD, (number of trials), SD was only calculated for n > 2

In an effort to increase the melting temperature a disulfide bond was added between FR2 and FR3 in order to create a double cysteine variant termed L1-G2+. The location of the cysteine substitutions was chosen analogous to the positions reported by Hagihara et al. [14], the first group to report on this method of disulfide bond addition in order to increase the melting temperature of a sdAb. Since that first report other examples of sdAb achieving an increased melting temperature with a similarly placed disulfide bond have been described [13, 15–19].

The L1-G2+ clone showed a melting temperature of ~80 °C while maintaining nM affinity (K_D_, see Table 2), however protein yields were reduced by at least 5-fold (3.9 mg/L). An additional undesirable effect due to addition of the second disulfide bond was that L1-G2+ lost its ability to refold (Table 1). This is in contrast to other sdAb with an added disulfide bond where we observed slightly improved refolding [19] and (Table 1).

**Table 2:** Binding kinetics

Clone	k_a_ (1/Ms)^a^ Average; SD	k_d_ (1/S)^a^ Average; SD	K_D_ (M)^a^ Average; SD
L1-G2	5.8 E+05; 6.4 E+04	6.3 E−04; 1.1 E−05	1.1 E−09; 1.7 E−10
L1-G2+	5.9 E+05; 1.1 E+05	1.3 E−03; 1.7 E−04	2.4 E−09; 6.6 E−10
L1-G2+neg	4.2 E+05; 3.9 E+04	1.4 E−03; 1.3 E−04	3.3 E−09; 2.0 E−10
L1-G2+neg2	5.4 E+05; 6.1 E+04	1.3 E−02; 3.5 E−04	2.3 E−08; 3.0 E−9

^a^Based on four measurements

One approach we took towards increasing the production of L1-G2+ was to have the sequence synthesized with its codons optimized for *E. coli* expression. Not surprisingly, the yields for the optimized version were essentially the same as those for the unmodified version. Although sometimes codon usage may play a limiting role in protein expression, the original high yields of the L1-G2 implied this was not likely the problem with the expression of the L1-G2+ variant.

**Fig. 1:**
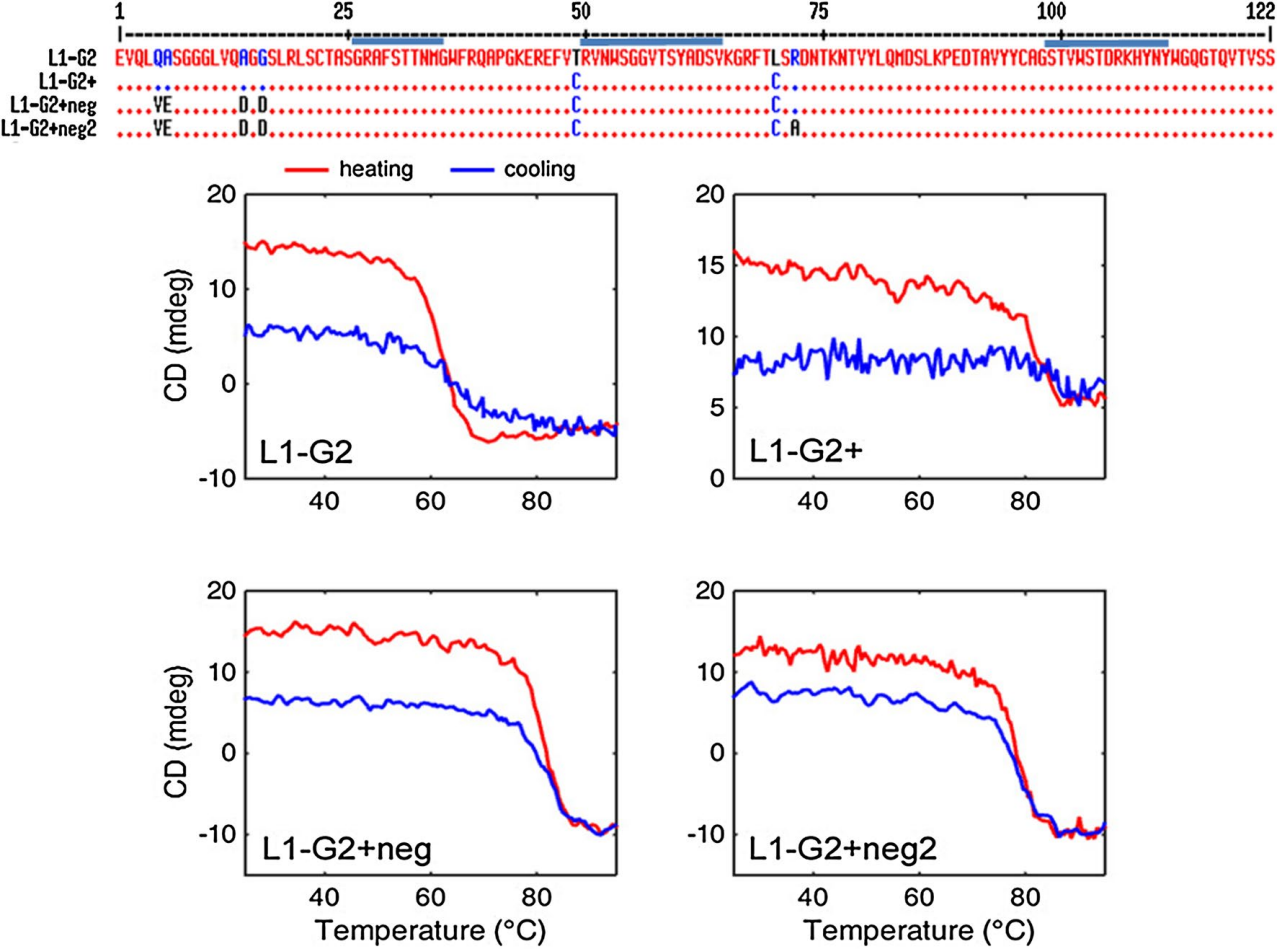
Sequence alignment using MultAlin [29] and CD heating and cooling curves. *Top panel* shows the sequence alignment of L1-G2, L1-G2+, L1-G2+neg, and L1-G2+neg2. The *blue bars* indicate the positions of CDR1, 2, and 3. The initial two amino acids (MA) and the amino acids added due to the restriction site and the His-tag are not show above (AAALEHHHHHH). The *bottom panels* show the melting and re-folding of the sdAb L1-G2, L1-G2+, L1G2+neg, L1G2+neg2 as measured by CD. Heating curves are shown in *red* and cooling in *blue*

Previously, we had showcased versions of ricin binding sdAb in which negative charges introduced through point mutations resulted in mutants with improved solubility and that refolded essentially 100 % [13]. Variants of three of these sdAb that contained the extra disulfide bond spanning the framework were also constructed. All showed an increased melting temperature, retained the ability to refold, and expressed in good quantity. In light of this observation, we hypothesized that the protein production of L1-G2+ might be restored by introducing additional negatively charged amino acids to lower the isoelectric point. Sequence comparisons, utilizing high melting temperature sdAb A3 and D12 isolated by our group, guided the choice of the mutations [13, 28]. This was accomplished by simply looking for acceptable negatively charged framework amino acids in these two highly stable sdAb and assuming that these changes would also be acceptable for the sdAb being modified. Based on this analysis, we constructed clone L1-G2+neg that included three negatively charged residues; the top panel of Fig. 1 shows the primary sequences of L1-G2, L1-G2+, and L1-G2+neg. Table 3 shows the number of charged residues for the various sdAb produced, and their calculated as well as measured pI. Interestingly, the calculated pI was generally one unit lower than that measured by isoelectric focusing (IEF, see Additional file 1: Figure S1). The bottom panel of Fig. 1 shows circular dichroism data from which melting temperature and refolding ability is determined. The L1-G2+neg mutant refolded better than the L1-G2+, while maintaining a melting temperature of 78 °C. Importantly, the average yield of L1-G2+neg was 13.8 mg/L, about 3.5 times better than the yield of L1-G2+. Each of the mutant proteins were prepared at least four separate times. The average yield of L1-G2+ over seven independent productions was 3.9 mg/L with a standard deviation of 1.1, and values ranged from 2.4 to 5.7 mg/L; yields of G2+neg ranged from 13.1 to 15.2 mg/L with an average of 13.8 and standard deviation of 1.4 over four independent trials (Table 1). Cultures for each independent protein production were started from separate colonies; at least two of the independent preparations were performed on different weeks.

**Table 3:** Number of charged residues, calculated/measured isoelectric point, and Tm for sdAb

Clone	Negative	Positive	pI (calc)^a^	pI (IEF)^b^
L1-G2+	10	13	8.8	>10.7
L1-G2+neg	13	13	7.0	8.3
L1-G2+neg2	13	12	6.6	7.7
A3+	12	12	7.0	8.2
A3+neg	14	11	6.0	6.8
AC+	11	11	7.2	8.2
AC+neg	12	11	6.6	7.7
AC+neg2	13	11	6.3	7.2

^a^Calculated using ExPASy ProtParam tool

^b^Measured by IEF see Additional file 2: Figure S2

**Fig. 2:**
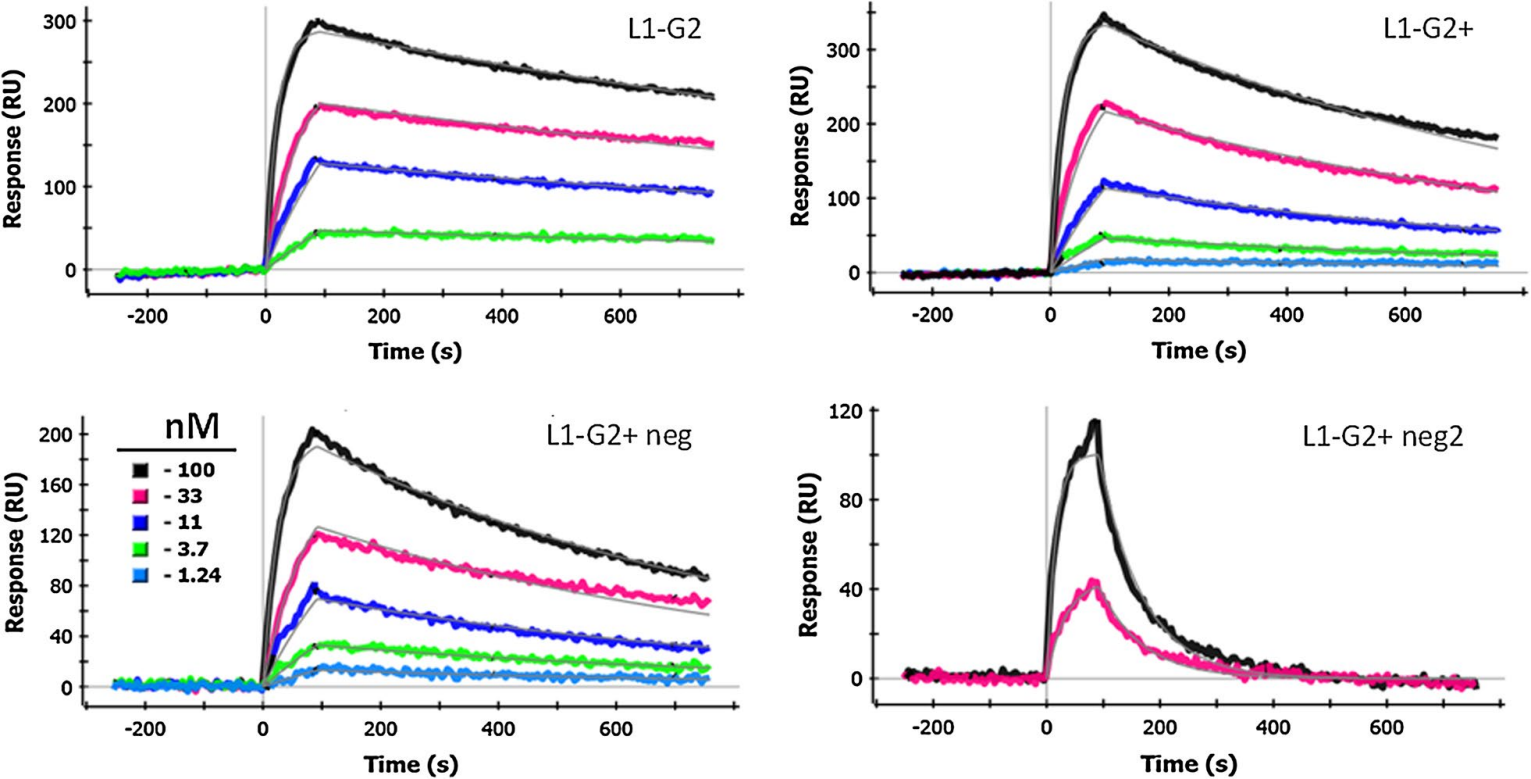
Representative SPR data and fits. In each case the L1 protein has been immobilized on the SPR chip and dilutions of the indicated antibody are flowed over the chip. For all the sdAb shown three-fold dilutions starting with 100 nM were tested, the *color* key is shown in the L1-G2+neg plot. All plots were interspot and blank corrected

While addition of the negative charges to the L1-G2+ clone substantially improved the protein production, just as important was to confirm the affinity of the L1-G2+neg to be similar to the parental L1-G2+ (Table 2; Fig. 2). We had previously determined that the off rate of L1-G2+ is higher than for the L1-G2 clone [18]; this was confirmed in the present measurements. We found the off rate of the L1-G2+neg to be the same as the L1-G2+, however a slightly slower on rate led to an overall dissociation constant of 3.3 versus 2.4 nM. We have found some variability of the kinetics depending on the antigen surface, thus we consider these affinities (differing less than a factor of 2) to be essentially the same.

In order to further increase the net negative charge of the L1-G2+neg sdAb, we further substituted an alanine for an arginine in the framework 3 section of the sdAb producing clone L1-G2+neg2 (top panel Fig. 1). The melting temperature for this construct is the same as for L1-G2+, and it showed ~78 % refolding according to CD experiments. Unfortunately the binding of L1-G2+neg2 was greatly decreased, showing an extremely fast off rate and a K_D_ of 23 nM versus 3.3 nM for the L1-G2+neg (Fig. 2; Table 2). Although there is no crystal structure for L1-G2 or its variants, by examining the recently published structure of a toxin binding sdAb [30], we postulate that the arginine may make interactions with CDR2; thus, its substitution likely caused an altered conformation of the binding region.

**Fig. 3:**
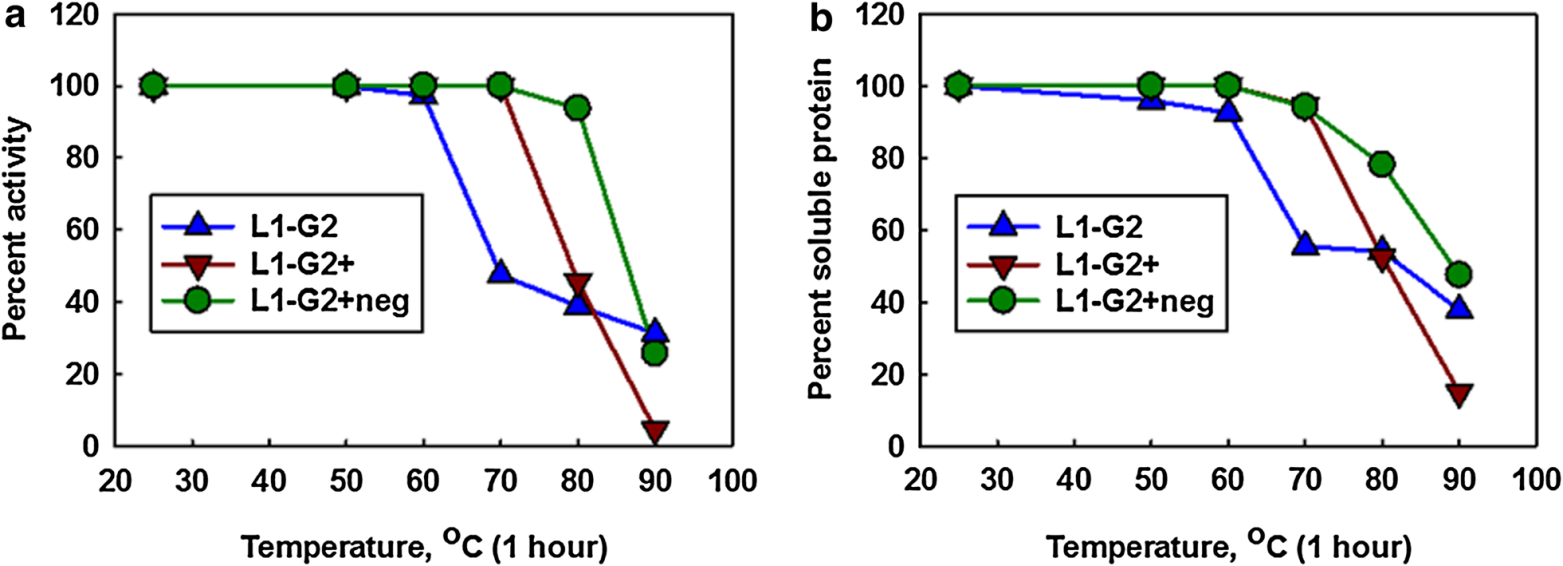
Retention of sdAb binding activity and solubility following heating. SdAb at a concentration of 1 mg/ml were heated for one hour at the indicated temperature. **a** The percent of binding activity retained was determined by initial on rates ascertained by SPR. The binding rate of unheated sdAb was set to 100 %. **b** Samples were spun and the OD at 280 nm was compared to unheated samples to determine the percent soluble protein. These are plots of representative data, the same trends were seen on replication

We subjected the L1-G2, L1-G2+, and L1-G2+neg constructs to a heat challenge in which each was incubated at six temperatures ranging from 25 to 90 °C for an hour at a concentration of 1 mg/mL. After incubation the solubility and activity of the samples, as assessed using surface plasmon resonance (SPR), was determined (Fig. 3). Essentially each sdAb retained close to 100 % of its activity up to its melting temperature, however upon exposure to temperatures above their melting temperatures the activity decreased. The L1-G2 lost activity between 60 and 70 °C, L1-G2+ lost activity between 70 and 80 °C, while L1-G2+neg did not start to lose activity until heating to 90 °C (Fig. 3a). Measurements of the percent soluble protein, determined by centrifuging each sample and recording the absorption of the samples after heating, followed the same trend as the activity determinations (Fig. 3b). Despite the ability of the L1-G2 and the L1-G2+neg to refold as demonstrated by CD, these proteins are apparently aggregating when heated at high concentrations for extended times. Our findings are in contrast to a report that found that chemical modification, rather than aggregation was responsible for permanent heat denaturation of sdAb [31]. This suggests that there may be no single universal mechanism of heat induced irreversible denaturation in sdAb.

In order to determine if decreasing the isoelectric point was a general method to restore protein production for sdAb with added disulfides we engineered negative mutations into two Staphylococcal enterotoxin B (SEB) binding sdAb, A3 and AC, that had been mutated to add a disulfide spanning frameworks 2 and 3 (termed A3+ and AC+). The A3 sdAb had a melting temperature of ~85 °C and could be produced at levels of ~11.5 mg/L, and while the melting point of A3+ increased to >90 °C, the yield dropped to an average of only ~1.7 mg/L. A version of A3+ with two added negative charges, termed A3+neg was constructed in order to improve production (Additional file 2: Figure S2). The A3+neg melted at ~87 °C and maintained its affinity for SEB, however, at ~2.3 mg/L, protein yields were only marginally higher than those of A3+. All three constructs, A3, A3+, and A3+neg, have essentially the same affinity (not shown), and regain the majority of their secondary structure after heat denaturation (Table 1).

Similar affinity and production results were obtained for the negative mutants of AC+. Whereas production of AC+ decreased by a factor of 7.6 versus AC (Table 1), the addition of negative charges only improved the diminished yield by ~30 %. In this case however, we also examined the impact of the negative charges on AC in the absence of the added disulfide bond. ACneg, in which we changed residues QA in frame work 1 to VE (Additional file 3: Figure S3), not only produced as well as the wild type AC, it’s melting point was 7 °C higher, equivalent to AC+’s. As VE is common to many of our most stable sdAb, we have potentially identified stabilizing framework residues that can be utilized to enhance a majority of sdAb; a finding that awaits verification.

One question that arises from this work is, “Why did addition of negative charges to L1-G2+ greatly improve production, while gains were marginal when the same approach was applied to A3+ and AC+?”. One of the differences between L1-G2+neg and A3+neg and AC+neg was that for L1-G2+, three negative charges were added, while for A3+neg, two negative charges were added and one positive charge removed and AC+neg, one negative charge added; the total number of charges for L1-G2+neg increased from 23 to 26, while A3+neg only increased from 24 to 25 total charges, and AC+neg only went from 22 to 23 charges (see Table 3). Thus, the charge structure of L1-G2+neg was changed more substantially than that of either A3+neg or AC+neg, which could be related to its improved production.

One might presume that it is the probability that an improper disulfide bond forms during the initial folding pathway that causes the production limitations of these disulfide mutants. Changing the amino acid sequence, especially neutral/charge changes or hydrophilic/hydrophobic changes, likely impacts the folding pathway probabilities, and thus, on occasion can decrease improper disulfide formation and thereby improve yields. Expression of additional DsbA and DsbC by utilizing plasmids such as pTUM4 to correct improperly formed disulfides has proven useful for production of proteins with multiple disulfide bonds [32]. Its utility will be investigated for these proteins in the near future.

## Conclusions

The production of stable, high affinity binding reagents is important for a variety of applications; however the reagents have to be well expressed in order for their utilization to be practical. The incorporation of negatively charged amino acids into the sequence of a version of the sdAb L1-G2 that was engineered to include an additional disulfide bond led to increased protein production and improved refolding without causing significant loss in melting temperature or affinity. However, this approach provided little benefit for two other sdAb possessing an additional disulfide bond. Thus, while disulfide bond addition appears to be a general method for sdAb stabilization, albeit often accompanied with impaired production; the addition of negative charges may or may not help restore production, but can still improve a variety of the sdAb biophysical properties: i.e. thermal stability, refoldability, and solubility.

## Methods

### Materials

The L1-G2, L1-G2+, A3, and A3+ sdAb were developed as previously described [18, 19]. Oligonucleotids, gene synthesis, and DNA sequencing were by Eurofins Genomics. The L1 reagent was obtained through BEI Resources, NIAID, NIH: Vaccinia Virus, Western Reserve, L1R Protein with C-Terminal Histidine Tag, Recombinant from baculovirus. Mutagenesis to create both the L1-G2+neg and A3+neg was performed using the Quick Change kit from Agilent Technologies. Sequence alignments were performed using MultAlin [29]. Chemicals were from VWR, or Sigma unless otherwise indicated.

### Protein preparation

Each protein was prepared at least two times; some were prepared as many as seven times. Cultures for each preparation were started from independent single colonies. At least two of the preparations were performed on different weeks with colonies always started from fresh transformations.

Protein was produced by following the protocol for periplasmic protein preparation described previously [11, 12, 18]. Briefly, we transformed Rosetta (DE3) with expression plasmids, and grew colonies overnight at 37 °C on LB agar plates with 100 µg/mL ampicillin and 34 µg/mL chloramphenicol. The next day 50 mL overnight cultures were started from single colonies and grown at 25 °C in terrific broth (TB) with 100 µg/mL ampicillin and 34 µg/mL chloramphenicol. The overnight cultures were used to inoculate larger cultures (500 mL of TB with 100 µg/mL ampicillin and 34 µg/mL chloramphenicol), which were grown for 3 h at 25 °C before expression was induced by addition of 0.5 mM isopropyl β-d-1-thiogalactoside (IPTG). After induction, cultures were grown an additional 2.5 h and then the cells were pelleted. Cell pellets were homogenized in 14 mL cold sucrose-tris (750 mM sucrose, 100 mM Tris pH 7.5), and then 28 mL of 1 mM ethylenediaminetetraaceticacid (EDTA; pH 8) was added drop-wise to each sample. The cells were shaken for 15 min on ice, and then 1 mL of 500 mM MgCl_2_ was added and the samples incubated on ice a further 10 min before pelleting the cells. Five millilitre of 10× IMAC buffer (0.2 M Na_2_HPO_4_, 4 M NaCl, 0.2 M imidazole, pH 7.5) and 0.5 mL of Ni Separose (GE Healthcare) were added to the supernatant and the sample tumbled at least 1 h at 4 °C on a rotisserie. Next, the resin was washed twice in batch with 25 mL 1× IMAC buffer. The resin was poured into a small column, washed with a further ~10 mL 1× IMAC buffer and eluted with 1 mL of 1× IMAC buffer containing 500 mM imidazole. Protein was then further purified into PBS by size exclusion chromatography using a GE Healthcare Superdex 75 10/300 GL column or Bio-Rad Enrich SEC70 column 10/300 and a Bio-Rad Duo-Flow System. Yield of the sdAb was determined by UV spectroscopy using a Nanodrop (Thermo).

### Purity and pI determination

The purity of a number of sdAb utilized in this work was confirmed by running samples diluted to 0.2 mg/mL on the Bio-Rad Experion system following the manufacturer’s protocol for reduced proteins. As expected each protein showed only a single major band with a molecular weight between 15 and 18 kDa, Additional file 4: Figure S4.

The isoelectric point of the majority of the mutants utilized in this manuscript were determined by isoelectric focusing (IEF). All mutants with a unique pI were tested, i.e. only AC+ was tested as representative of the pI for both AC and AC+. The IEF was performed using 5 % polyacrylamide Novex native IEF gels and buffers according to manufacturer’s protocol (Life Technologies).

### Circular dichroism

As described previously, a Jasco J-815 CD spectrometer was utilized to determine the melting temperature and refolding ability of the sdAb [11, 18, 19]. Samples were diluted into deionized water to a final concentration of 40 µg/mL. As the temperature was increased from 25 to 90 °C at a rate of 2.5 °C/min, the differential absorbance of the sdAb sample was measured at 208 nm. The melting point correlated to the temperature at the inflection point between the folded and unfolded state. The error on the melting point determinations is within ±1 °C. For several of the constructs, replicate protein preparations were analyzed by CD and showed essentially the same melting and refolding behavior. Dye melt determinations were performed as described previously [13, 19].

### Surface plasmon resonance

A Bio-Rad ProteOn XPR36 system was used to assess binding kinetics of the sdAb to target (L1 or SEB) immobilized on channels of a standard GLC sensor chip as detailed previously [18, 19]. Briefly, the antigen was immobilized to the sensor chip surface on four rows at saturation concentrations of 10 µg/mL using EDC/NHS chemistry. The chip was turned 90 degrees, then dilutions of each sdAb (ranging from 100 to 0 nM) were flowed across the chip for 120 s at 100 µL/min, and the association was recorded. Next, dissociation was monitored as buffer was flowed over the chip for 600 s. The L1 surface was regenerated flowing through 50 mM glycine (pH 2.5) between individual samples. The one shot kinetics were determined from each of the antigen coated rows using five concentrations of single domain antibody, and kinetic parameters were calculated using the standard Langmuir binding model available on the ProteOn Manager RM 2.1 software (Bio-Rad). Typically the four values for K_D_ are within 20 %, and the range of values from the four measurements was always within a factor of 2.

## Additional files

**Additional file 1: Figure S1.** Isoelectric focusing gel of sdAb. Measurement of isoelectric point using isoelectric focusing gel electrophoresis. Each well was loaded 10 µg of sample, except 4 µg of lane 5 sample was loaded. Lanes 1 and 10 represent the Serva pI marker (pH 3-10 from Life technologies Inc). Lane 2: Ac+neg: lane 3: AC+neg2; lane 4: AC+; lane 5: A3+; lane 6: A3+neg; lane 7: G2+; Lane 8: G2+neg; Lane 9: G2+neg2.
**Additional file 2: Figure S2.** Sequence alignment of sdAb A3 and variants. Sequence alignment of the SEB binding sdAb A3, A3+, and A3+neg using MultAlin [29]. The initial two amino acids (MA) and the amino acids added due to the restriction sites and the His-tag are not show above (AAALEHHHHHH).
**Additional file 3: Figure S3.** Sequence alignment of sdAb AC and variants. Sequence alignment of the SEB binding sdAb AC, AC+, AC+neg and AC+neg2 using MultAlin [29]. The initial two amino acids (MA) and the amino acids added due to the restriction sites and the His-tag are not show above (AAALEHHHHHH).
**Additional file 4: Figure S4.** Molecular weight and purity assessment of sdAb. Figure S4. Assessment of purity for purified single domain antibodies on gel electrophoresis. The virtual gel was obtained from Experion Pro260 chip (Bio-Rad laboratories). Approximately 200 µg/mL for each protein sample was used. The peak density of purified single domain antibodies as indicated by the blue arrow is >95 %. The rest of the bands represent high and low markers and internal systematic bands as indicated by the magenta arrows and described as such in the manufacturer’s protocol (Bio-Rad). Sample order is as follows, Lane L: Molecular marker Ladder. L1: ACneg; L2: AC+neg; L3: AC+neg2; L4: AC+;L5: A3+; L6: A3+neg; L7: G2+; L8: G2+neg; L9: G2+neg2; L10: G2.
