# Supplementary material for: Enhanced production of a single domain antibody with an engineered stabilizing extra disulfide bond
Source: Microb Cell Fact. 2015 Oct 9;14:158. doi: 10.1186/s12934-015-0340-3 (PMC4599338; doi:10.1186/s12934-015-0340-3)
Supplement: Supplementary file 1 — Additional file 1: Figure S1. Isoelectric focusing gel of sdAb. Measurement of isoelectric point using isoelectric focusing gel electrophoresis. Each well was loaded 10 µg of sample, except 4 µg of lane 5 sample was loaded. Lanes 1 and 10 represent the Serva pI marker (pH 3-10 from Life technologies Inc). Lane 2: Ac+neg: lane 3: AC+neg2; lane 4: AC+; lane 5: A3+; lane 6: A3+neg; lane 7: G2+; Lane 8: G2+neg; Lane 9: G2+neg2. [file 12934_2015_340_MOESM1_ESM.pdf]

Additional file 1: Figure S1. Isoelectric focusing gel of sdAb.

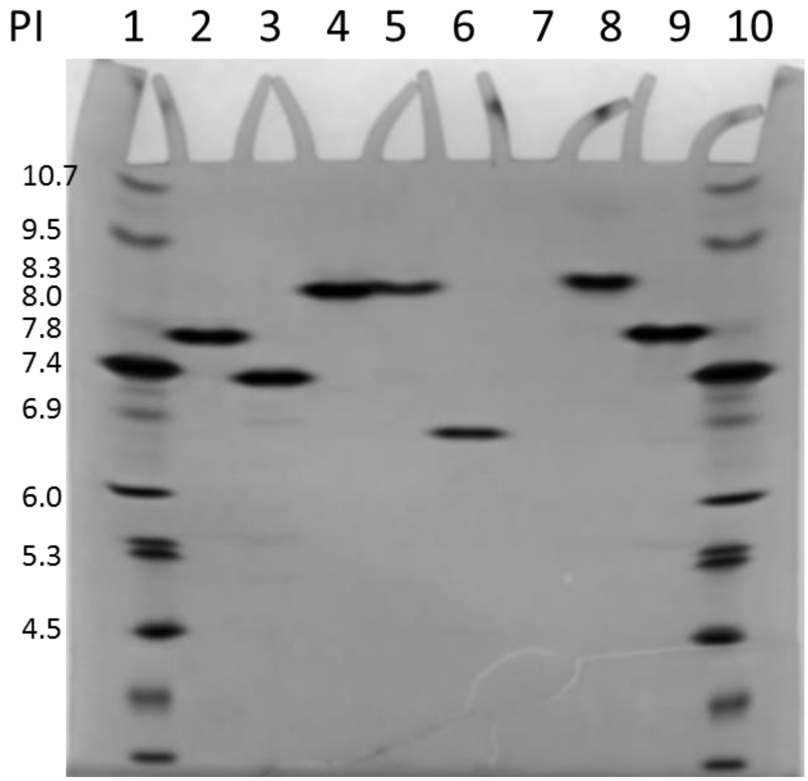

Figure S1. Measurement of isoelectric point using isoelectric focusing gel electrophoresis. Each well was loaded 10 µg of sample, except 4 µg of lane 5 sample was loaded. Lanes 1 and 10 represent the Serva pI marker (pH 3-10 from Life technologies Inc). Lane 2: Ac+neg: lane 3: AC +neg2; lane 4: AC+; lane 5: A3+; lane 6: A3+neg; lane 7: G2+; Lane 8: G2+neg; Lane 9: G2+neg2.
