# Supplementary material for: Enhanced production of a single domain antibody with an engineered stabilizing extra disulfide bond
Source: Microb Cell Fact. 2015 Oct 9;14:158. doi: 10.1186/s12934-015-0340-3 (PMC4599338; doi:10.1186/s12934-015-0340-3)
Supplement: Supplementary file 2 — Additional file 2: Figure S2. Sequence alignment of sdAb A3 and variants. Sequence alignment of the SEB binding sdAb A3, A3+, and A3+neg using MultAlin [29]. The initial two amino acids (MA) and the amino acids added due to the restriction sites and the His-tag are not show above (AAALEHHHHHH). [file 12934_2015_340_MOESM2_ESM.pdf]

Additional file 2: Figure S2. Sequence alignment of sdAb A3 and variants.

```
         1       10        20        30        40        50        60        70        80        90        100       110       120      129
         |--------+---------+---------+---------+---------+---------+---------+---------+---------+---------+---------+---------+--------|
     A3  EVQLVESGGGLVQAGDSLRLSCTASGRTFSRAVMGWFRQAPGKEREFVAAISAAPGTAYYAFYADSVRGRFSISADSAKNTVYLQMNSLKPEDTAVYYCAADLKMQVAAYMNQRSVDYWGQGTQVTVSS
    A3+  ................................................C.......................C........................................................
A3+neg   .............D................D.................C.......................C........................................................
```

Figure S2. Sequence alignment of the SEB binding sdAb A3, A3+, and A3+neg using MultAlin [32]. The initial two amino acids (MA) and the amino acids added due to the restriction sites and the His-tag are not show above (AAALEHHHHHH).
