# Supplementary material for: Enhanced production of a single domain antibody with an engineered stabilizing extra disulfide bond
Source: Microb Cell Fact. 2015 Oct 9;14:158. doi: 10.1186/s12934-015-0340-3 (PMC4599338; doi:10.1186/s12934-015-0340-3)
Supplement: Supplementary file 3 — Additional file 3: Figure S3. Sequence alignment of sdAb AC and variants. Sequence alignment of the SEB binding sdAb AC, AC+, AC+neg and AC+neg2 using MultAlin [29]. The initial two amino acids (MA) and the amino acids added due to the restriction sites and the His-tag are not show above (AAALEHHHHHH). [file 12934_2015_340_MOESM3_ESM.pdf]

Additional file 3: Figure S3. Sequence alignment of sdAb AC and variants.

```
        AC  DVQLQASGGGLVQPGGSLRLTCAASGLIFGSYAMGWFRQAPGKAREFVAAISWSGGDTYADSVKGRFTISRDNAKNTVYLQMNSLEPEDTAVYSCAAVGSKYYISKDAKDYGYWGQGTQVTVSS
       AC+  ................................................C...................C.......................................................
    AC+neg  ....VE..........................................C...................C.......................................................
   AC+neg2  ....VE..........................................C...................C.......................................................E
```

Figure S3. Sequence alignment of the SEB binding sdAb AC, AC+, AC+neg and AC+neg2 using MultAlin [32]. The initial two amino acids (MA) and the amino acids added due to the restriction sites and the His-tag are not show above (AAALEHHHHHH).
