# Supplementary material for: Enhanced production of a single domain antibody with an engineered stabilizing extra disulfide bond
Source: Microb Cell Fact. 2015 Oct 9;14:158. doi: 10.1186/s12934-015-0340-3 (PMC4599338; doi:10.1186/s12934-015-0340-3)
Supplement: Supplementary file 4 — Additional file 4: Figure S4. Molecular weight and purity assessment of sdAb. Figure S4. Assessment of purity for purified single domain antibodies on gel electrophoresis. The virtual gel was obtained from Experion Pro260 chip (Bio-Rad laboratories). Approximately 200 µg/mL for each protein sample was used. The peak density of purified single domain antibodies as indicated by the blue arrow is >95 %. The rest of the bands represent high and low markers and internal systematic bands as indicated by the magenta arrows and described as such in the manufacturer’s protocol (Bio-Rad). Sample order is as follows, Lane L: Molecular marker Ladder. L1: ACneg; L2: AC+neg; L3: AC+neg2; L4: AC+;L5: A3+; L6: A3+neg; L7: G2+; L8: G2+neg; L9: G2+neg2; L10: G2. [file 12934_2015_340_MOESM4_ESM.pdf]

Additional file 4: Figure S4. Molecular weight and purity assessment of sdAb.

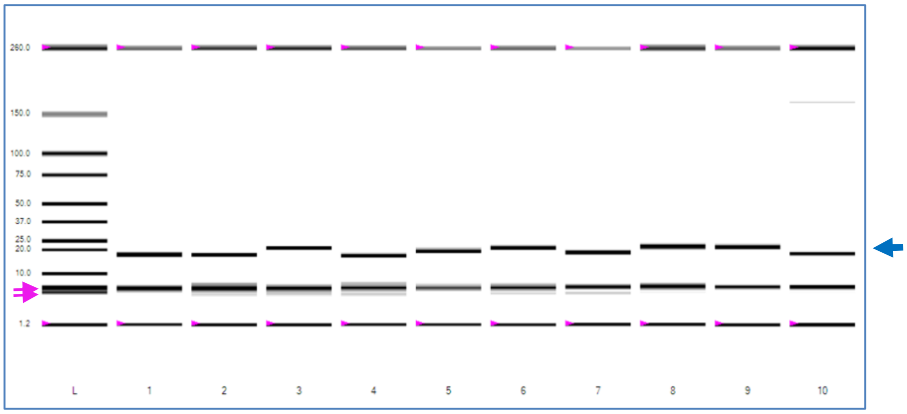

Figure S4. Assessment of purity for purified single domain antibodies on gel electrophoresis. The virtual gel was obtained from Experion Pro260 chip (Bio-Rad laboratories). Approximately 200 µg/mL for each protein sample was used. The peak density of purified single domain antibodies as indicated by the blue arrow is >95%. The rest of the bands represent high and low markers and internal systematic bands as indicated by the magenta arrows and described as such in the manufacturer’s protocol (Bio Rad).

Sample order is as follows, Lane L: Molecular marker Ladder. L1: ACneg; L2: AC+neg; L3: AC+neg2; L4: AC+;L5: A3+; L6: A3+neg; L7: G2+; L8: G2+neg; L9: G2+neg2; L10: G2.
